# Supplementary material for: Modifying Electrochemical Doping in Light‐Emitting Electrochemical Cells With Gold Nanoparticles
Source: Adv Sci (Weinh). 2026 Oct 4:e78120. Online ahead of print. doi: 10.1002/advs.78120 (PMC13634572; doi:10.1002/advs.78120)
Supplement: Supplementary file 1 [file ADVS-9999-e78120-s001.pdf]

SUPPORTING INFORMATION

Modifying Electrochemical Doping in Light-Emitting Electrochemical Cells with Gold Nanoparticles

Ajay K. Poonia[1, 2, 3*], Anton Kirch[2], Joan Ràfols-Ribé[1, 2], Lucrezia Catanzaro[4], Anish Rao[5], Karol Kołątaj[5], Vittorio Scardaci[4], Giuseppe Compagnini[4], Guillermo P. Acuna[5], Ludvig Edman[2, 3*], and Nicolò Maccaferri[1, 3*]

[1]Ultrafast Nanoscience Group, Department of Physics, Umeå University, Umeå, 901 87, Sweden

[2]The Organic Photonics and Electronics Group, Department of Physics, Umeå University, Umeå, 901 87, Sweden

[3]Wallenberg Initiative Materials Science for Sustainability, Department of Physics, Umeå University, Umeå, 901 87, Sweden

[4]Department of Chemical Sciences, University of Catania, Catania, 95125, Italy

[5]Department of Physics, University of Fribourg, Fribourg, 1700, Switzerland

**ajay.poonia@umu.se*

**ludvig.edman@umu.se*

**nicolo.maccaferri@umu.se*

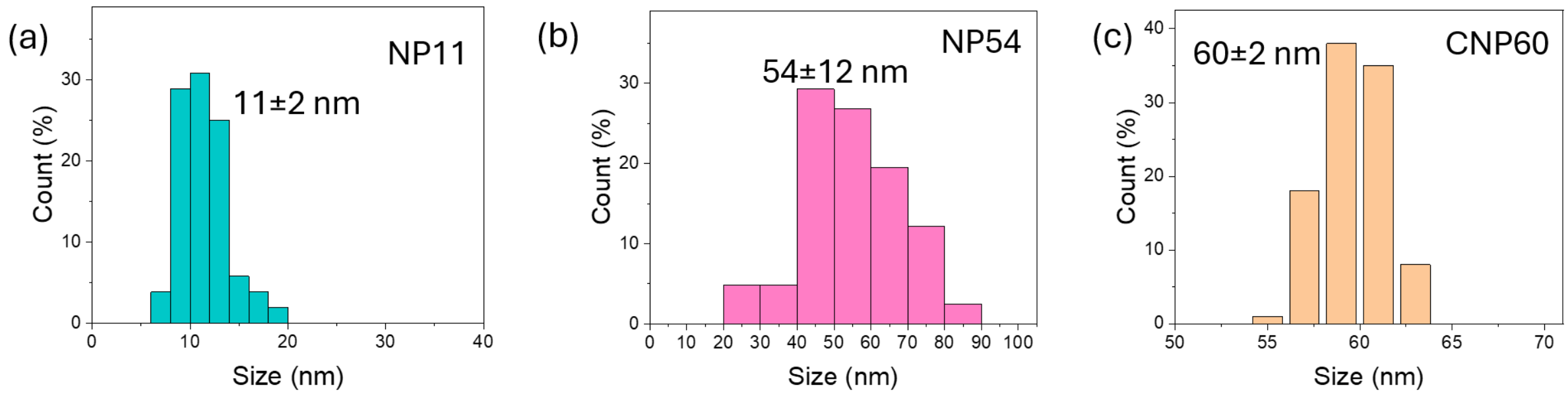

Figure S1. Size distribution of Au NPs, obtained by TEM measurements for (a) NP11, (b) NP54, and (c) CNP60.

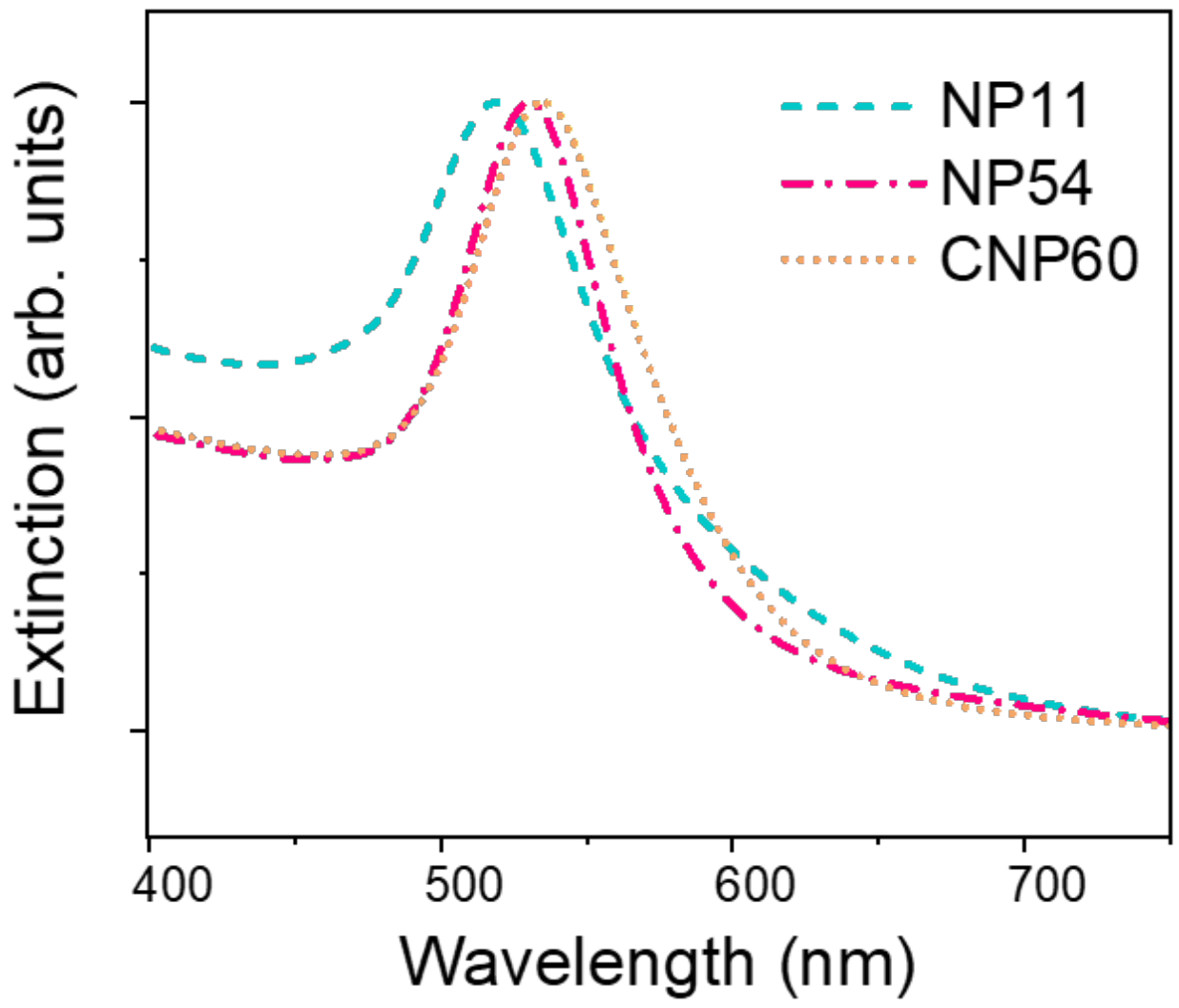

Figure S2. Extinction spectra of Au NPs in the solution.

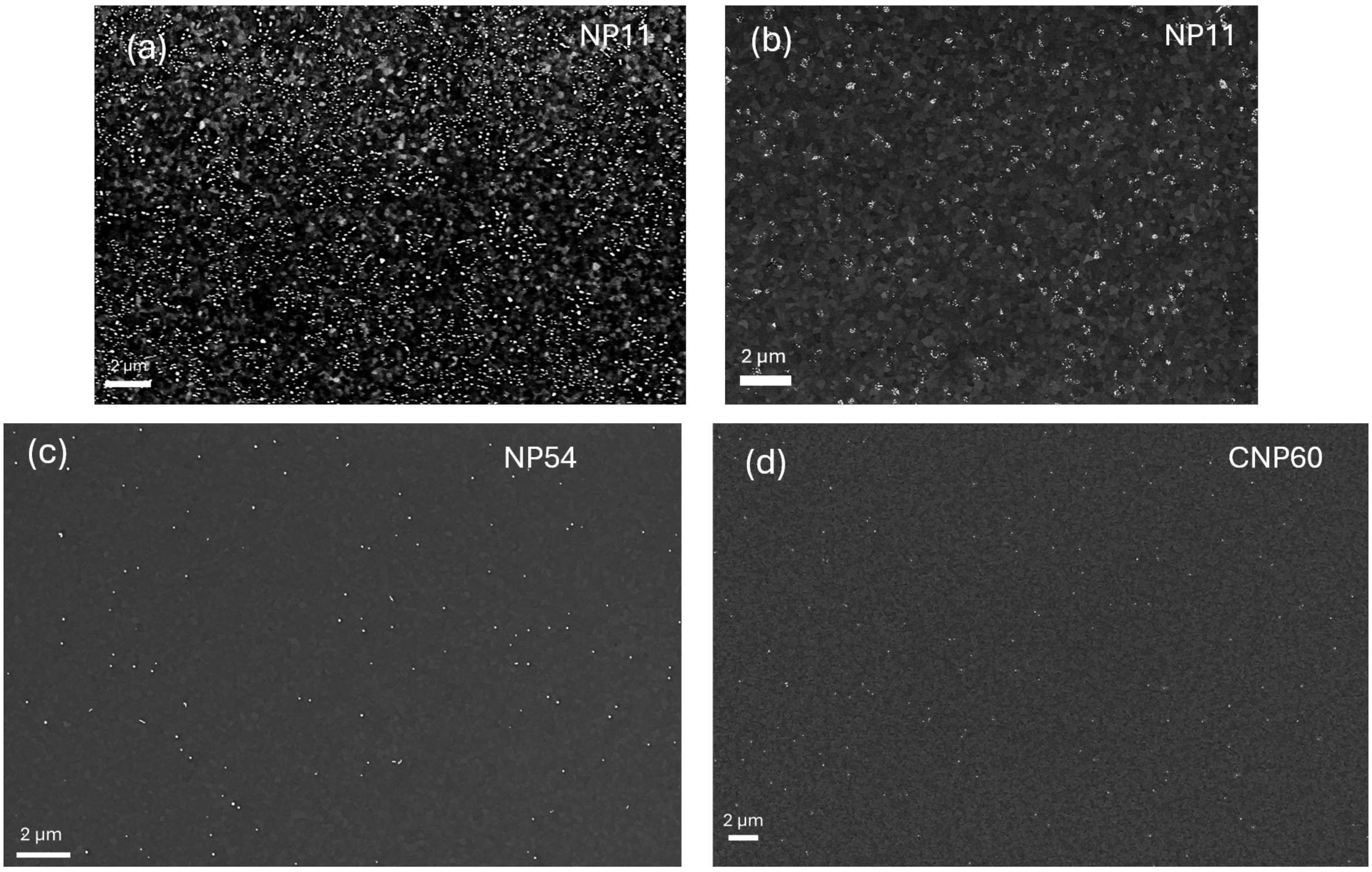

Figure S3. SEM images of spin coated NPs (white dots) on ITO coated glass substrate: (a) NP11 with surface coverage of ~12 %, (b) NP11 with surface coverage of ~1 %, (c) NP54 with surface coverage of ~ 0.2%, and (d) CNP60 with surface coverage of ~ 0.2%.

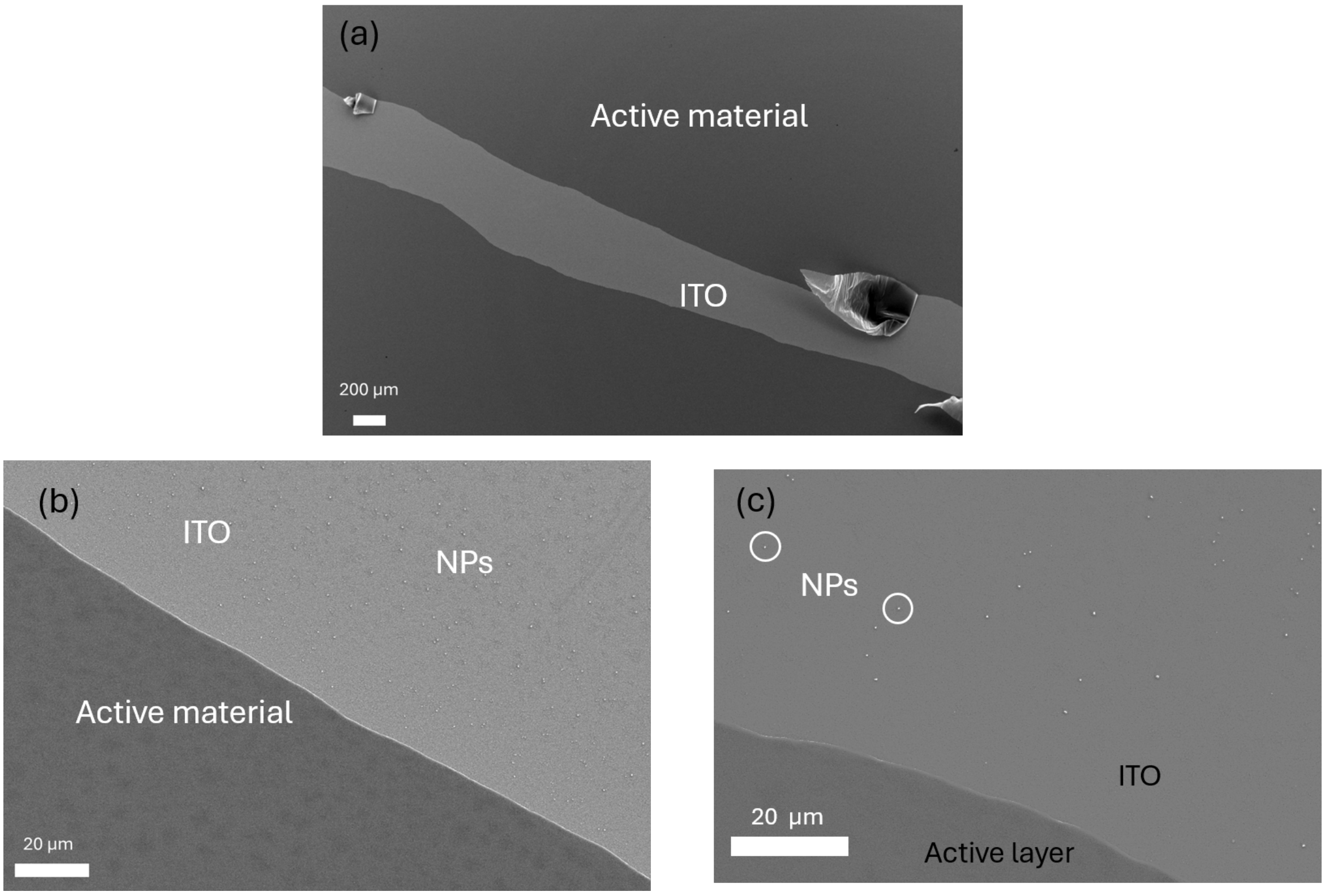

Figure S4. (a) SEM image of AM layer coated on NP11 deposited ITO substrate, AM layered peeled off from the center of the image. (b, c) Zoomed SEM images showing NPs (while dots) on top of ITO.

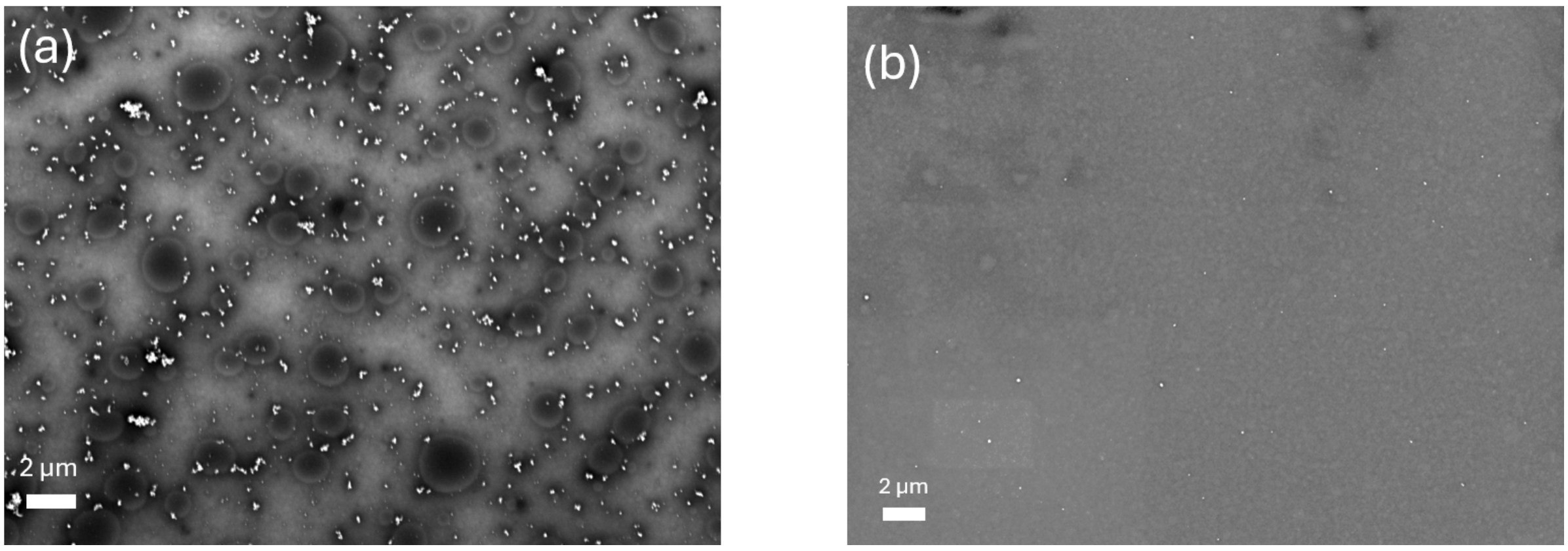

Figure S5. SEM image of NPs deposited on an ITO substrate after coating and subsequent removal of the AM layer with cyclohexane, confirming the presence of NPs following AM layer coating (a) NP11 (b) NP54.

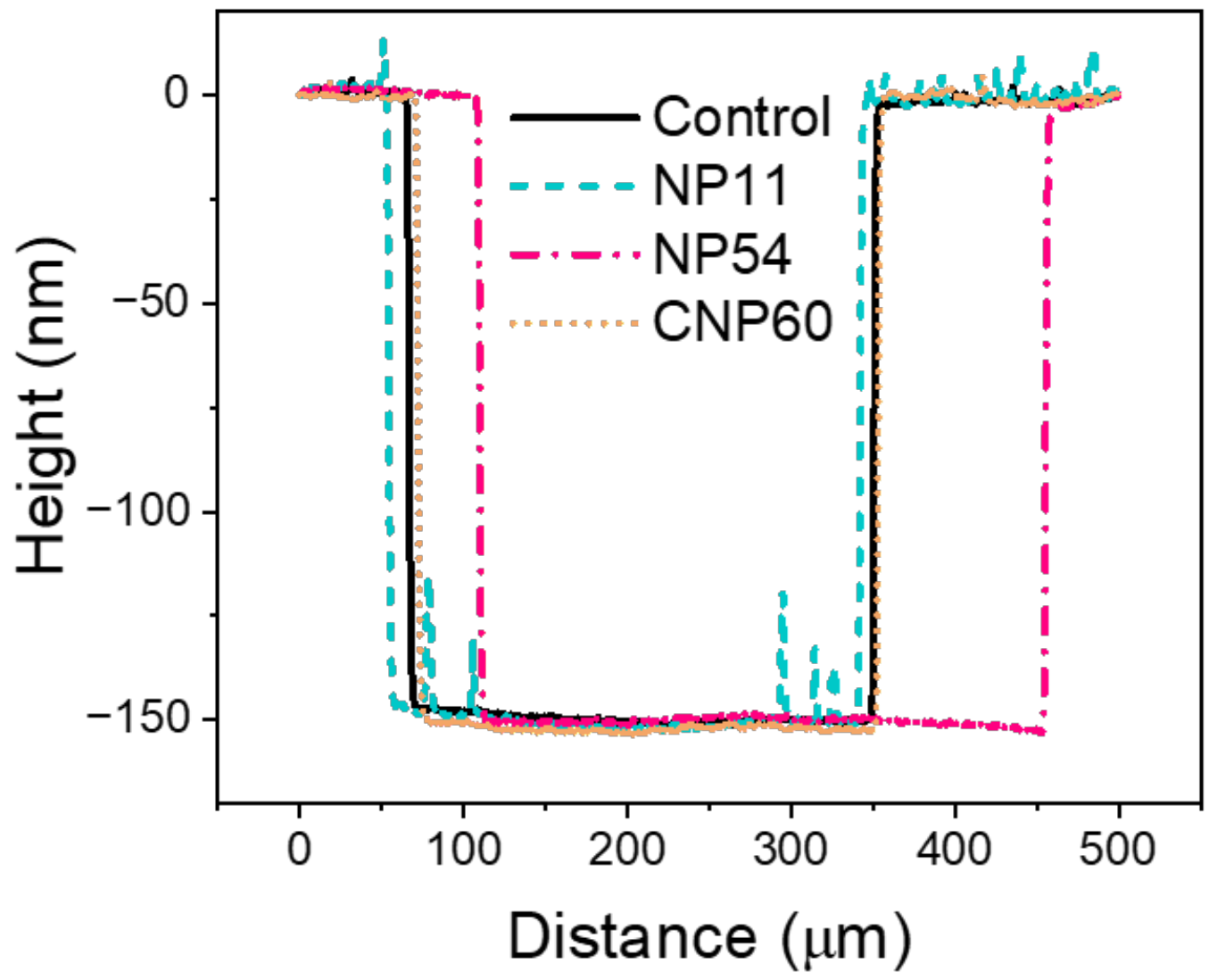

Figure S6. The active-layer thickness of control and NP-LECs.

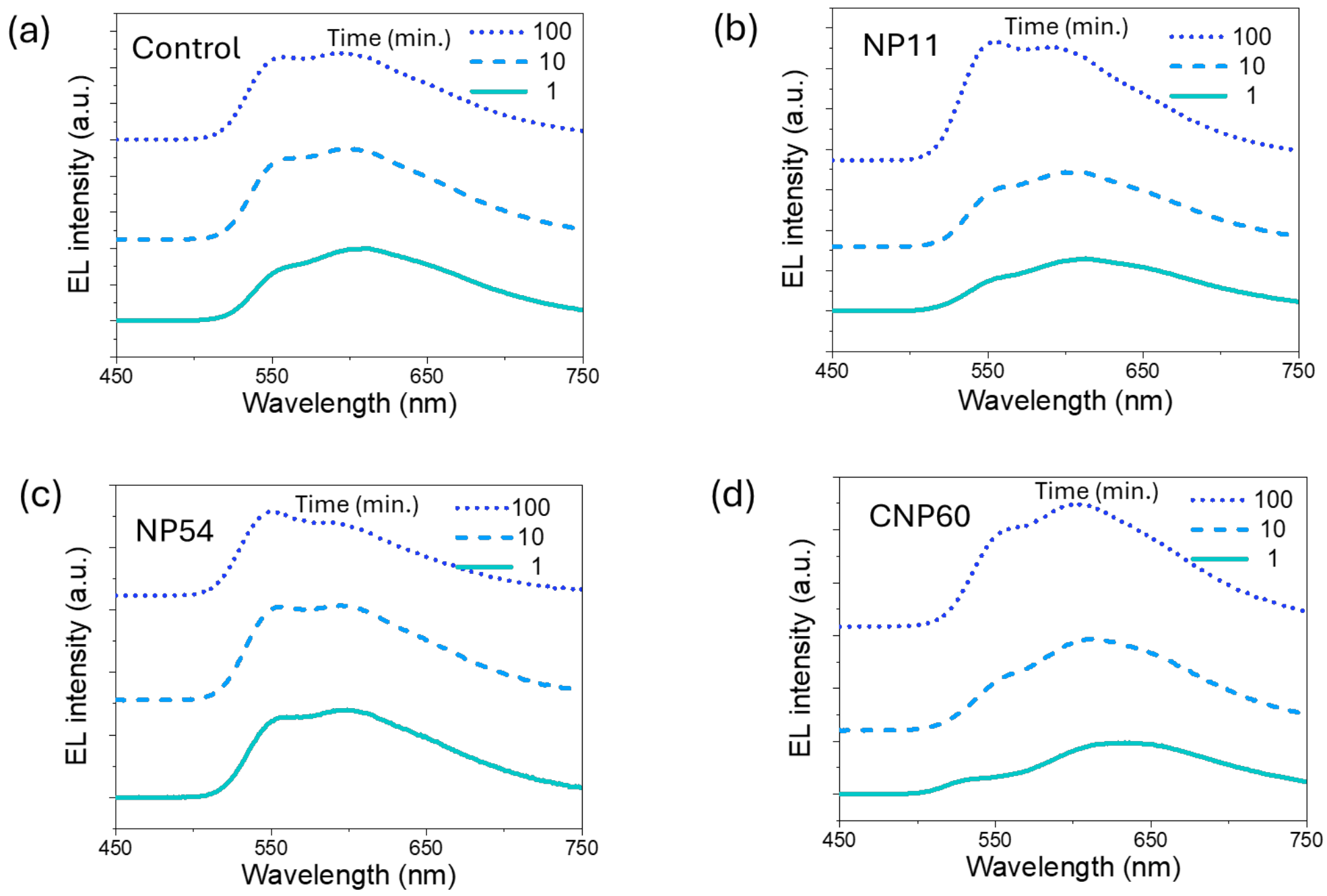

Figure S7. EL cross section at selected times (a) control-LEC (b) NP11-LEC (c) NP54-LEC (d) CNP60-LEC, showing distinct spectral profiles for control- and NP-LECs corresponding to different EZ positions.

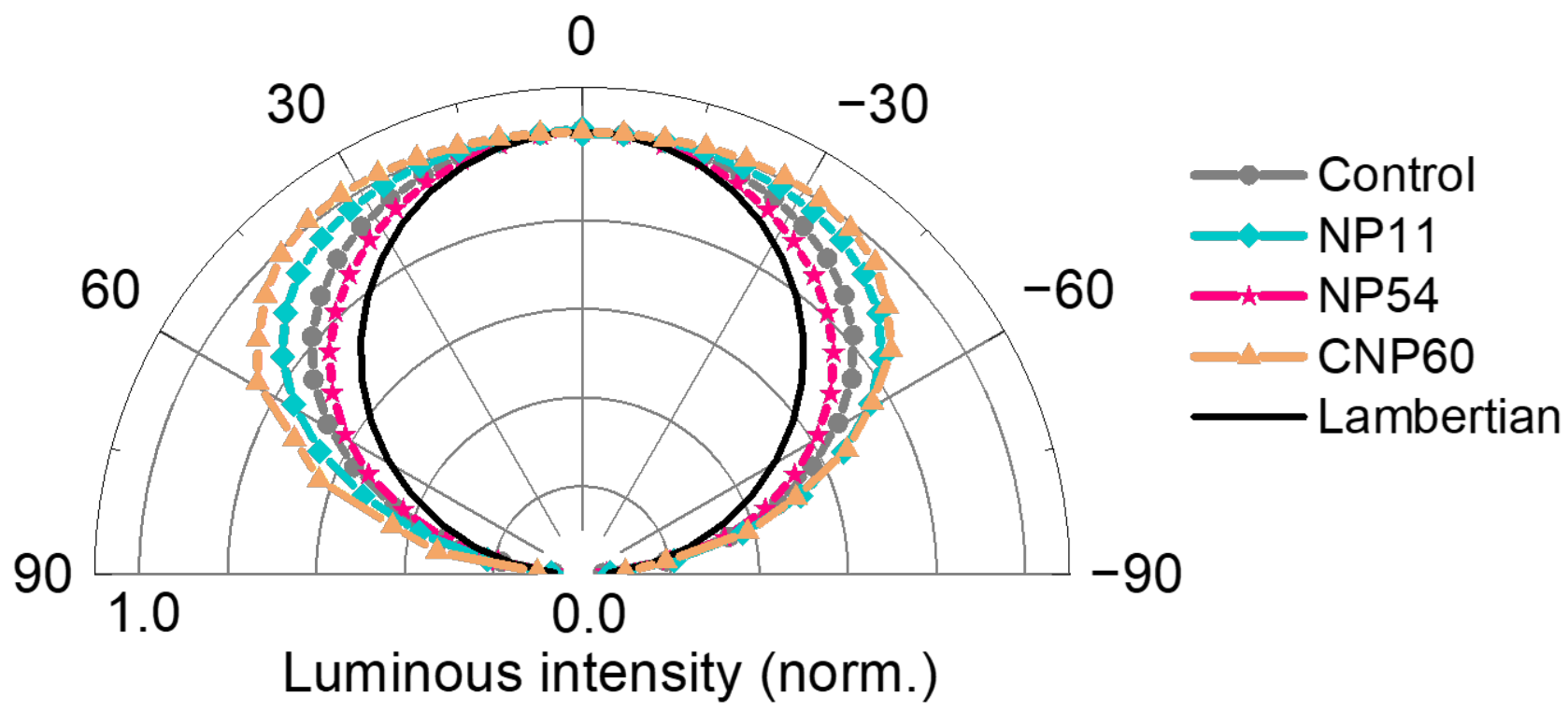

Figure S8. Polar plots of normalized EL intensity versus emission angle for control- and NP-LECs in the early stage of operation (at t= 10 min).

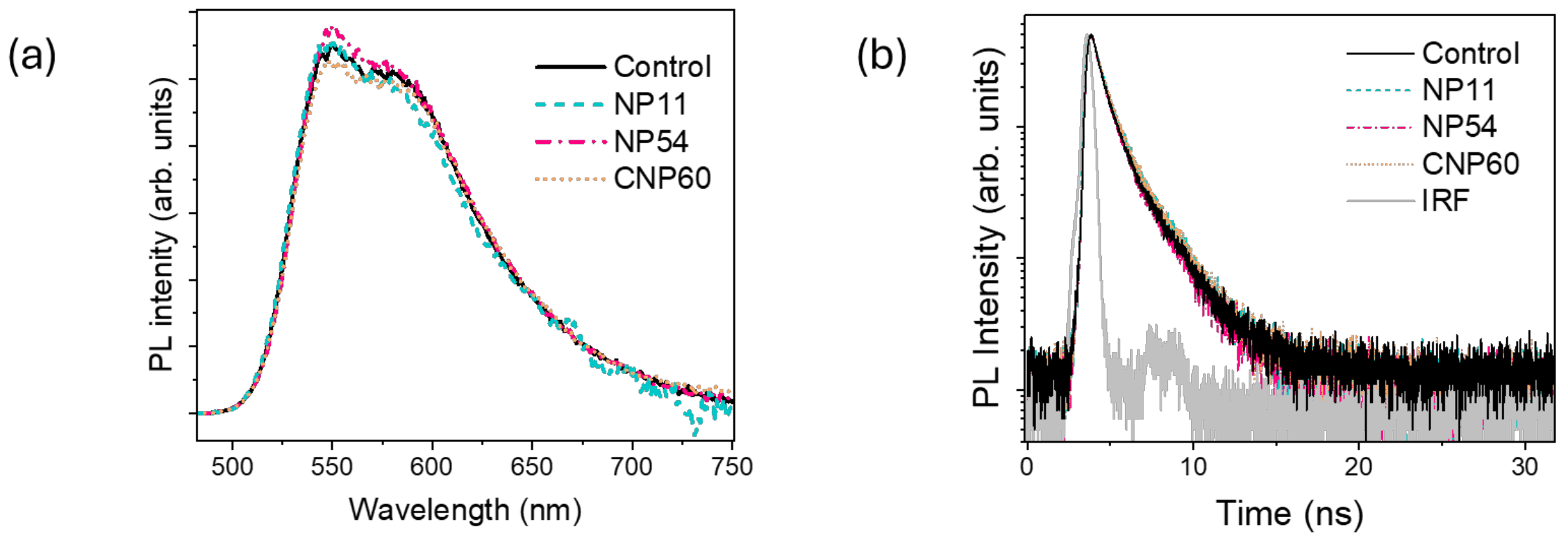

Figure S9. (a) Steady state photoluminescence of control and NP-LECs (b) Lifetime of photoluminescence (measured with time-correlated single-photon counting (TCSPC) spectroscopy) for control and NP-LECs.

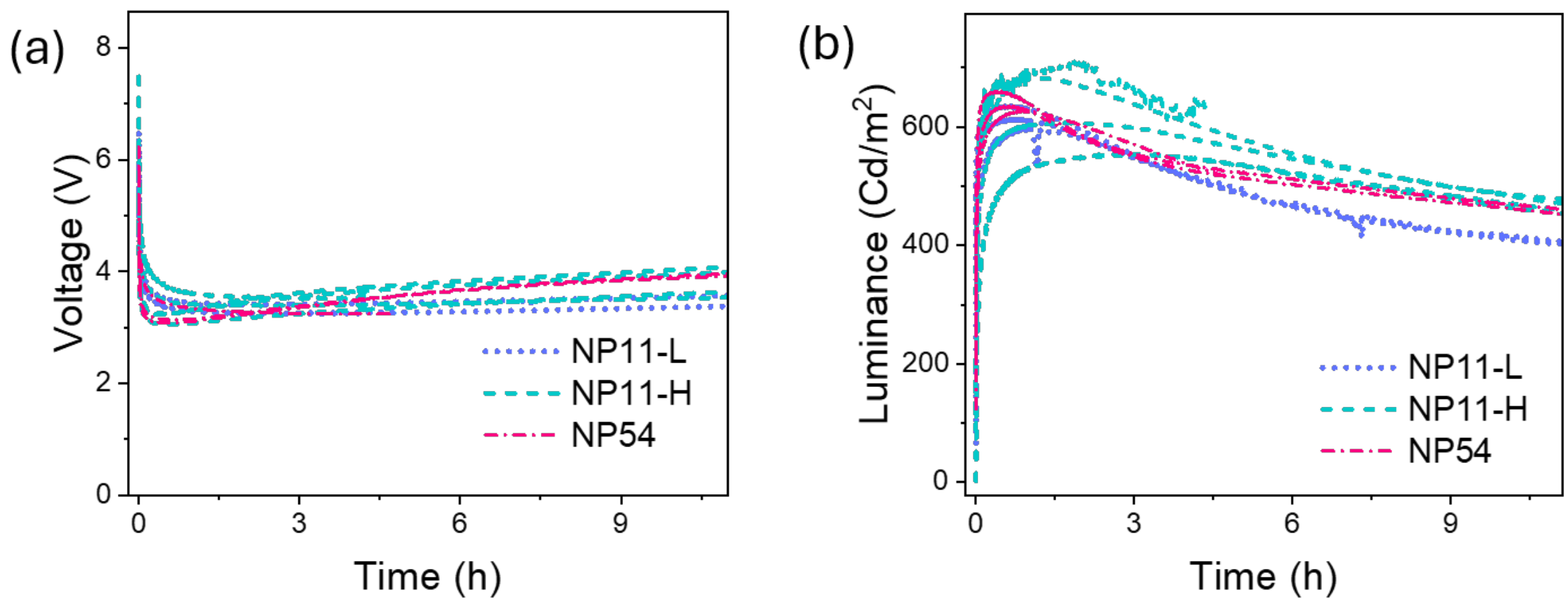

Figure S10. The time evolution of (a) the voltage and (b) the luminance for the NP11 LECs with low (L) and high (H) surface coverage of NP11, driven with a constant current density of 25 mAcm^{-2}.

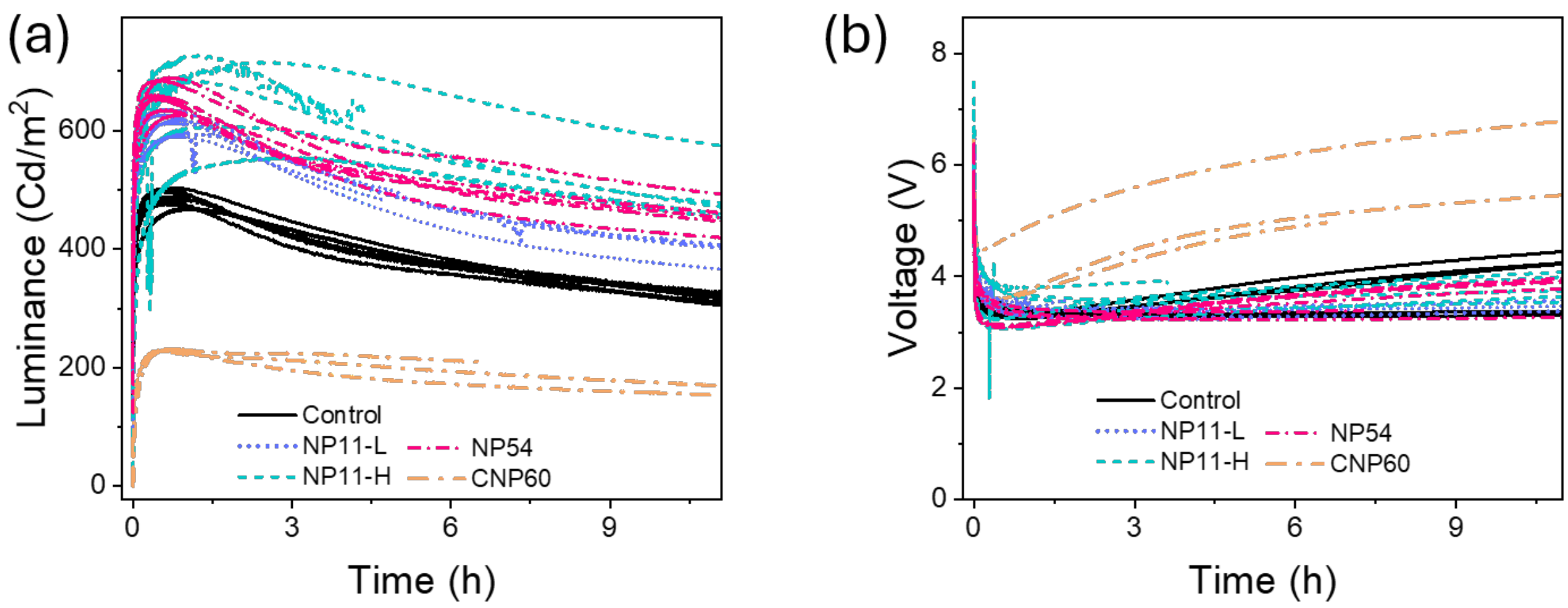

Figure S11. Time evolution of (a) voltage (b) luminance for control- and NP-LECs driven by under a current density of 25 mA cm^{-2}. (Here NP11-H and NP11-L represent LEC devices with surface coverage of 12% and 1%, respectively).
